# Norwegian nursing and medical students’ perception of interprofessional teamwork: a qualitative study

**DOI:** 10.1186/1472-6920-14-170

**Published:** 2014-08-14

**Authors:** Ingunn Aase, Britt Sæthre Hansen, Karina Aase

**Affiliations:** 1 Department of Health Studies, University of Stavanger, Stavanger N-4036, Norway; 2 Department of Health Studies, University of Stavanger and Stavanger University Hospital, Stavanger N-4036, Norway

**Keywords:** Interprofessional teamwork, Interprofessional education, Professional role, Content analysis, Healthcare, Students’ perceptions

## Abstract

**Background:**

Little is known about the ways in which nursing and medical students perceive and understand their roles in interprofessional teamwork. A 2010 report by the World Health Organization highlights the importance of students’ understanding of teamwork in healthcare, and their ability to be effective team players. This study aims at describing nursing and medical students’ perceptions of interprofessional teamwork, focusing on experiences and recommendations that can be used to guide future educational efforts.

**Methods:**

The study uses a qualitative research design. Data were collected from four focus group interviews: two homogenous groups (one with medical students, one with nursing students) and two mixed groups (medical and nursing students).

**Results:**

The results show that traditional patterns of professional role perception still prevail and strongly influence students’ professional attitudes about taking responsibility and sharing responsibility across disciplinary and professional boundaries. It was found that many students had experienced group cultures detrimental to team work. Focusing on clinical training, the study found a substantial variation in perception with regard to the different arenas for interprofessional teamwork, ranging from arenas with collaborative learning to arenas characterized by distrust, confrontation, disrespect and hierarchical structure.

**Conclusions:**

This study underlines the importance of a stronger focus on interprofessional teamwork in health care education, particularly in clinical training. The study results suggest that the daily rounds and pre-visit “huddles,” or alternatively psychiatric wards, offer arenas suitable for interprofessional training, in keeping with the students’ assessments and criteria proposed in previous studies.

## Background

Interprofessional teamwork in healthcare has gained increasing recognition worldwide as a way to increase patient safety [1] and to foster collaborative and effective teams e.g., [2,3]. The World Health Organization (WHO) has highlighted the importance of interprofessional teamwork and recommended educational programs that equip health care students with the necessary skills and competence to become effective team players [1,4].

International research [5-7] corroborates the position taken by the WHO, but studies also reveal difficulties in implementing interprofessional educational efforts [2,3,5,8] and suggest that undergraduate education largely fails to address key elements, such as the understanding of professional roles, authority, hierarchy and gender related dimensions of teamwork [1,2,7,9].

Of interest for the current study is the fact that Norwegian authorities have taken steps to promote interprofessional teamwork and education. The National Health Plan [10] acknowledges interprofessional collaboration as a critical element for ensuring quality in health care services. In a White Paper submitted to the Norwegian Parliament [11], the Ministry of Education sets requirements for the inclusion of interprofessional teamwork in health education. Reviewing lessons learned from the Norwegian initiatives, Clark [12] concluded that the emerging positive outcomes have been somewhat impaired by lack of resources.

Previous studies by Kyrkjebø *et al*. [13] and Bjørke [14] noted that Norwegian students are not sufficiently exposed to interprofessional teamwork during their clinical training. Other Norwegian studies reported similar results [15,16]. Aase *et al.*[8] found that theoretical lectures on interprofessional teamwork were not followed-up in clinical training, especially in nursing schools. Medical schools exposed their students to more interprofessional training, but still fell short of full compliance with the WHO recommendations [8]. The reasons for this are partly because of structural constraints, such as resources, and partly because of faculty and students’ attitudes [12].

Saroo *et al.*[17] argue that successful interprofessional training should take advantage of the students’ psycho-sociological determinants, such as professional role behavior, hierarchy, and power relations.

Based on this information, we surmise that a thorough understanding of the students’ perspective is imperative for designing successful interprofessional training. The current study analyses data from focus group interviews with nursing and medical students who had been exposed to interprofessional teamwork during their clinical training in Norway. Grounded in the students’ perceptions, the analysis aims at describing patterns and recommendations for the design of future interprofessional training. Note that the qualitative framework allowed the students to include reflections on the group processes – i.e., the focus group interviews – that were part of the current study.

## Conceptual background

Interprofessional teamwork is discussed by Reeves *et al.*[9] who stated that the concept implies common goals, shared team identity, shared commitment, clear team roles and responsibilities, interdependence between team members, and integration between work practices [pp. 3–4]. West *et al.*[18] concluded that clear professional roles are essential, and that team members may benefit from a comprehensive understanding of both their own professional role and the professional roles of their colleagues. Petri [19] suggested that interprofessional teamwork is best attained through an education that promotes mutual trust and respect, effective and open communication, and the awareness and acceptance of the roles, skills, and responsibilities of participating disciplines. Damour and Oansan [20] noted that educational efforts should be marshalled early in the curriculum, prior to the solidifying of professional identities and the formation of stereotypes.

Some authors have suggested that interprofessional teamwork requires strong collaborative skills that are not included in the training of health professionals [2,21]. Others have hypothesized that the lack of attention to interprofessional teamwork in educational programs may reflect an expectation that professionals will intuitively know how to work collaboratively [9].

## Methods

### Design

This study used a qualitative design, using focus groups as a vehicle for acquiring the viewpoints of many respondents in a short period of time. The hallmark of focus group interviews is that interaction among participants tends to stimulate richer or deeper expressions of opinion [22]. The reporting of the methodology of this study follows the RATS (Relevance, Appropriateness, Transparency, Soundness) guidelines for qualitative studies.

Based on data obtained from four focus group interviews with nursing and medical students in Norway, this study was guided by two research questions:

•What are students’ perceptions of their professional roles in the context of interprofessional teamwork?

•How do students perceive interprofessional teamwork arenas?

The term *arena* is used here to denote the setting and occasion underpinning team work, for example, ward rounds. The questions were grounded in our goal to conceptualize students’ perceptions to guide the design of future interprofessional training. The first research question was constructed to focus on professional roles that have been reported to have a strong effect on interprofessional teamwork [e.g., 9]. Assuming the students would easily recognize professional role behavior, we focused on this rather than on more abstract concepts.

The second question directs attention towards arenas with the potential for interprofessional teamwork, assuming these venues may serve as bases for future training.

Defining the main themes of the current study, the research questions were used by the group facilitator – the second author – who steered the discussion to maintain focus. The research questions also guided the structure of the focus groups.

### Pilot testing of questions

To ensure that the students would adequately understand the questions in the interview guide, pilot interviews were conducted with two groups of nursing students. These students were excluded from the ensuing research interviews.

### Context

The data were collected during the clinical training period of 42 medical students and 180 nursing students at a university hospital in Norway in the autumn of 2011. Medical and nursing students were enrolled in study programs at two separate universities.

### Participants

The demographic data are shown in Table 1. The nursing and medical students were comparable in terms of their gender distribution, and the nursing students tended to be somewhat older.

**Table 1:** Demographic data

**N 22**	**Nursing students**	**Medical students**
Participants	10	12
Gender	6 female, 4 male	7 female, 5 male
Age	20-29 years: 6	20-29 years: 12
	30–39 years: 4	30–39 years: 0
Clinical practice experiences includes clinical training in education	1-3 years: 5	1-3 years: 4
	4–6 years: 2	4–6 years: 7
	7–9 years: 0	7–9 years: 1
	10–15 years: 3	10–15 years: 0

When we invited participants, we selected students with a certain amount of clinical training and who had been exposed to interprofessional teamwork in their clinical training. These criteria led us to invite medical students in the seventh semester and nursing students in their fifth semester. A web site for educational institutions was used to invite the nursing students to participate in the study. The medical students were invited through their supervisor at the hospital. The four groups were composed as follows:

Group 1 Homogenous group of seven medical students.

Group 2 Homogenous group of four nursing students.

Group 3 Mixed group of three medical and four nursing students.

Group 4 Mixed group of two medical and two nursing students.

Owing to practical constraints, Groups 2 and 4 only contained four participants each. Although small, we found that these groups still elicited a broad range of ideas and comments. Data on participants’ age, gender, educational program, clinical practice, and professional experience were recorded.

### Data collection

Each group was interviewed once (four interviews altogether) in sessions that lasted about 1 hour. The interviews were conducted by two researchers to make reliable observations and avoid “moderator dominance” [22,23]. After the fourth group interview had been conducted, the recorded data showed little variation and as new information was not identified, the interview process was discontinued [23].

Field notes and a reflective diary were used to capture observations and non-verbal information during the focus group sessions. Audiotaped recordings of each group session were transcribed and analyzed prior to undertaking the next group interview. An interview guide was developed to guide the researchers and interviewers. The guide was modified after each interview session to focus on areas requiring further exploration and inquiry.

### Data analysis

The analysis was designed to capture textual content related to the research questions based on the transcribed text [24]. The resulting material was subsequently combined into one text that was subject to the researchers’ scrutiny and qualitative content analysis [24]. “Meaning units” (i.e., groups of words or phrases reflecting similar content and context) were identified, condensed and coded. The coded data were organized into sub-themes and aggregated into themes that reflected the content of professional roles and interprofessional teamwork, as summarized in Tables 2 and 3[24]. Following Polit and Beck [22] and Graneheim and Lundman [24], a process of collaborative analysis - engaging all of the authors to reduce subjective bias - was adopted to enrich reflection on the data and interpretations of them. The analysis ended when saturation of content and themes was achieved [22,24].

**Table 2:** Theme and Subthemes within “Responsibility in Professional Roles”

**Theme**	**Responsibility in professional roles**		
**Sub-themes**	Taking responsibility	Sharing responsibility	Avoiding responsibility

**Table 3:** Theme and subthemes within “Use of Interprofessional Arenas”

**Theme**	**Use of interprofessional arenas**		
**Sub-themes**	Collaboration and learning	Status quo	Frustration

### Ethical issues

No ethical issues were identified. The study was approved by the University of Stavanger, Head of department, Department of Health Studies, and by the University of Bergen, Vice Dean of Research, Faculty of Medicine and Dentistry, as well as the Norwegian Social Science Data Service (NSD) [No 28383]. Since no patients or patient information was involved, the study did not require an approval from the Norwegian Regional Committees for medical and health research ethics. The participants were asked to sign an informed consent form prior to the interviews.

## Results

The analysis identified two major themes that resonated across all four groups, which were labeled “*Responsibility in professional roles*” and “*Use of interprofessional arenas.*” While the overlap with the research questions is seen in the terms “professional roles” and “arenas,” the concepts of “responsibility” and “use of” emerged from the coding and should be considered grounded in the data.

The following sections present both main themes, and the corresponding sub-themes, which are summarized in Tables 2 and 3. We also describe findings pertaining to the group *processes*—i.e., the focus group interviews—that were conducted as part of this research study.

### Responsibility in professional roles

The coding introduced three subthemes: *taking responsibility*, *sharing responsibility* and *avoiding responsibility* (see Table 2) that were subsumed under the main theme: *Responsibility in professional roles*. The data strongly affirmed that the students’ education influenced their professional understanding of and relation to responsibility.

#### Taking responsibility

Medical students explained that a manifest and clear role expectation was conveyed to them during theoretical lectures and clinical training. Referred to as elite students, their importance and grave responsibility were highlighted from day one and continually thereafter. The students mainly perceived their medical education as being designed to produce General Practitioners (GPs) who were expected to work individually and not in teams. Hence, the educational program stressed, according to students’ assertions, the importance of individual determination, including an aptitude for taking responsibility and driving decision-making.

A medical student stated:

The program has a clear focus on what is expected of many of us; we have to deal with things there and then, and we have to spend much of the time alone. (Medical student 1)

Asked to comment on the capabilities of nurses, the medical students revealed a lack of knowledge, having little or “no knowledge of nursing education”. Unaware that the nursing students had been trained to measure blood pressure, some of the medical students explained that they were prepared to do the measurements themselves rather than asking for a nurse’s assistance.

Despite this ignorance, a few medical students had experience in medical programs that attempted to bridge the knowledge gap between the professions, resulting in the introduction of a “Follow a Nurse” program. A medical student commented:

A video “Follow a Nurse” shows what nurses do through a working day, how many patients they are responsible for, what expectations they have to the education and clinical training, as well as to themselves and their future colleagues. (Medical student 4)

#### Sharing responsibility

Contrary to the medical students, several nursing students expressed that they had a perception of being encouraged, both in theoretical lectures and in clinical training, *to share responsibility* while working in teams. They described their function as “the glue” that organized teamwork around the patient, a function that often required nurses to perform various tasks overlooked or neglected by other team members, tending to force nurses into a “handyman” type of role. The coordinating function apparently conferred a sense of cross-disciplinary and shared responsibility upon the nurses, suggesting that the underlying student statements should be classified under the subtheme *sharing responsibility*.

A nursing student commented:

I feel that we as nurses are doing a bit of everything; we are dealing with issues that are left behind by other professionals. (Nursing student 7)

A medical student expressed:

In the ward, one notices immediately that the nurses are coordinating everything around the patient. We ask the nurses if we need information. (Medical student 3)

Some nursing students experienced themselves as being complementary and supportive to the physicians, in a collaboration bolstered by a sense of shared responsibility. Responsible for measuring vital signs and preparing observational data sheets as well as other materials, the nursing students had noted that the physicians used and relied on the information, thereby reinforcing an impression that the nurses’ role was an important and necessary one. A nursing student said:

In clinical training, I appreciated collaborating with the physician when he took me seriously and I understood that what I prepared was really important to him. (Nursing student 2)

A medical student acknowledged that responsibility for communication with the patients, could sometimes benefit from being shared with nurses:

If the physician is incompetent to speak with patients, the nurses do the talking. They are good at it. If the patient lacks courage to speak with the physician, they can ask the nurse to do it. (Medical student 8)

#### Avoiding responsibility

A number of students made comments that were classified under the subtheme *avoiding responsibility*. Inadequate understanding of professional roles, unclear communication mixed with intimidation, fear and insecurity were factors that fueled *avoidance of responsibility,* according to the students. These assessments were articulated mostly in statements made by nursing students, but also by some medical students who reported distress and insecurity in hierarchical situations dominated by senior physicians or nurses.

A nursing student stated:

I do not know what is right to do when the nurses and the physicians are arguing, it is in many ways scary. I get insecure when they are blaming each other. I hope it never happens to me. (Nursing student 10)

A medical student noted:

Some of the senior physicians are really strict; I fear asking him if I am in doubt of something and when I am working in a new ward, some of the “old nurses” can be quite rude, saying “as a medical student you should know this.” (Medical student 8)

Some nursing students had been given advice to refrain from taking part in discussions:

In clinical training, I learned to follow orders from the physicians, and some of my supervisors recommended me not to voice my own opinions if “that physician” asked for special arrangements. (Nursing student 2)

Both student groups found that nurses deferred to physicians. Several nursing students recalled that they had given up their chairs to physicians, to let the physician have a better view of what was being presented. Such patterns of servility were perceived by some nursing students as detrimental to their role as team members.

A medical student had noted that the nurses’ attitude might not be welcomed by the physicians:

I think there are many nurses behaving as if the physicians are exalted and elevated above themselves. I am not certain that the physicians want this role. (Medical student 5)

### Use of interprofessional arenas

The students’ experiences with existing interprofessional arenas varied widely in clinical training. The analysis elicited three subthemes termed *learning and collaboration, status quo* and *frustration* (see Table 3). The student assessments highlighted that the teamwork they had experienced was strongly affected by the arenas through ward culture and administration.

Some wards maintained several arenas for interprofessional interaction, such as wards rounds, pre-visits (“huddles”), shared working areas, joint computer resources and, intermittently, common lunches. Others were more limited, and the interprofessional arenas were in many cases limited only to the ward rounds.

There was little focus on existing interprofessional arenas in the theoretical lectures.

#### Collaboration and learning

The students experienced wards with a favorable culture that students described as being characterized by the term “mutual respect.” Professionals on these wards actively used interprofessional arenas, for example ward rounds, to facilitate *collaboration and learning*. Some students described staff on these wards as role models, and enjoyed collaborating with them.

Feeling they were treated as valuable members of the team, many nursing students described the wards at psychiatric hospitals as favorable arenas for interprofessional teamwork. A nursing student elaborated:

In the psychiatric ward, my voice does count. There, the physicians and nurses ask me about patients’ situations, what I have done together with the patients and what I think will help the patients. (Nursing student 12)

The same applied to some degree to rehabilitation wards.

In general, several students recommended ward rounds as arenas for educational efforts, such as courses, targeting interprofessional teamwork. A nursing student expressed:

Ward rounds may be a good arena for learning interprofessional teamwork, since both nurses and physicians jointly meet the patients together there, and we can learn from our supervisors how our own profession communicates both with patients and other professions in a real situation. (Nursing student 9)

Some students suggested orchestrating training in interprofessional teamwork early in the students’ educational plan, contending that that would give the students a more “solid basis” for future collaborative work. Others pointed out that the timing would have to be balanced against other activities prioritized in the curriculum.

#### Status quo

Some wards were perceived by the students as “*old fashioned and status quo*” and “*hierarchical characterized by silo thinking*”. Physicians showed little interest in other professions’ tasks and capabilities. The students also observed that experienced nurses and physicians worked together in inflexible and traditional structures, following their own entrenched procedures regardless of whether new guidelines existed. Nursing students experienced little debate between professions, even in cases where disagreement regarding treatment and care obviously prevailed. The nurses preferred to confront the physician after the rounds in a more informal setting, or not at all.

Having experienced disparities between the day and afternoon shifts, some students contended that nurses and physicians appeared to collaborate better with less pressure during the afternoon shifts. Night shifts could not be discussed because of lack of experience among the participants in the current study.

The statements captured under *status quo* revealed that the majority of the students had few arenas for practicing teamwork skills. When discussing suggestions to train collectively, a group of students mentioned AHLR (acute heart lung resuscitation), or “ward rounds” as potential scenarios for training. Moreover, some medical students expressed a need for guidelines on how to conduct ward rounds:

*Nobody ever told me how to do ward rounds. And what are they for: updating the nurses or the physician? Is the patient the focus? Nobody ever told me. The ward rounds represent the few minutes a day the patient has with the physician.(Medical student 8)*

#### Frustration

A group of students described certain wards as arenas where the prevailing communication style was unpleasant and disrespectful to the hospital staff, students, and patients. Expressions of these concerns were categorized under the sub-theme *frustration*. As one medical student stated:

It’s really up to each physician. For example, if they are very confrontational during the pre-visit. Some physicians have confidence in nurses. Others do not and demonstrate this by making fools of the nurses or finding other ways to be unpleasant. You can really feel this in the atmosphere of the ward. (Medical student 6)

Perceived as an important parameter, the chief physician’s communication style was raised as a concern in a number of statements. Some chief physicians failed to prioritize supervising or even having discussions with students. Nursing students, in particular, misconstrued this behavior, seeing it as a request for them to remain “invisible” by refraining from commenting and actively engaging in the situation. The physicians, in turn, misunderstood the quiet nurses, assuming they were difficult to deal with.

In some wards, the only arena for interprofessional teamwork was the ward rounds. According to some students, this was sometimes because of the infrastructure. A nursing student pointed out:

Infrastructure and the buildings do not facilitate collaboration. We have separate working areas, the informal conversation and the informal interprofessionality are not present, and we have no designated meeting rooms. (Nursing student 11)

Both student groups described a lack of attention to interprofessional teamwork in their education: “We have little theoretical lecturing in interprofessional teamwork and interprofessional communication.”

A medical student described participation in a course in communication:

The course was limited to one specific arena and not defined as a learning activity with evaluation and learning outcomes all the way through our clinical training. The course was never mentioned again by our supervisors and teachers … what was the intention? (Medical student 2)

In contrast to the nurses, who appeared to be able to communicate more personally and emotionally with the patients, the medical students were reluctant and even somewhat frightened of revealing too much about themselves in “in-depth” conversations, even if they claimed to be committed to the well-being of their patients.

Some of the medical students admitted being concerned about their future positions demanding leadership skills; stating that nobody had taught them how to become good leaders.

### The group process

The focus groups of the current study were in themselves recognized as arenas for interprofessional collaboration by the participants. This section presents findings pertaining to the functioning of the focus group interviews rather than the students’ experiences in clinical training.

Several of the students expressed their appreciation for the focus groups, emphasizing the insight they had gained into each other’s roles and work tasks.

A medical student (from one of the mixed student group) summarized his view as follows:

These focus groups are an excellent arena for learning to know each other as human beings and as professionals. The group discussion made me realize that I would benefit from learning about interprofessional teamwork during a ward round. (Medical student 10)

However, several students pointed out that the discussions in the homogenous groups suffered from lack of knowledge about the profession not represented. The missing information was to some degree substituted by guesses and stereotypes. Contrarily, the mixed group discussion was characterized by more mutual interest and respect, according to the students.

## Discussion

The aim of this study was to conceptualize students’ perceptions of interprofessional teamwork, seeking to describe patterns and recommendations that may guide the design of future interprofessional training. The results showed that nursing and medical students perceived responsibility differently; the nursing students were more inclined to share responsibility than the medical students, who regarded taking responsibility more as an individual obligation.

The use of interprofessional arenas varied broadly from promoting collaboration and learning, to maintaining entrenched workflows (status quo), and finally to discourage collaboration in a manner perceived as frustrating.

### Role perception

The results presented in this study suggest that traditional patterns of professional role understanding reported in previous studies (Manias *et al.*[25] and Fougner *et al.*[26]) are still prevalent among medical and nursing students—in medical and nursing schools, as well as in clinical practice. Zaccagnini *et al.*[27] argued that role identification and clarity are necessary ingredients to empower nurses to work in interprofessional teams. Yet, there is little evidence to support the notion that role identity alone is a sufficient factor for effective interprofessional team performance. Notably, several medical students with a strong awareness of role identity, perceived themselves as reluctant to share responsibility, which is arguably a fundamental pillar of teamwork. The findings presented here, in keeping with the emphasis on mutual respect, cross-disciplinary communication and knowledge bridging the gap between professions, lead us to hypothetically suggest that a more balanced relationship between professional role identities, conferring a more similar sense of expectations and responsibilities, may be key to building effective interprofessional teams.

A finding of particular interest to the design of future training, is that both student groups expressed lack of knowledge about each other’s roles and responsibilities which, in many cases, led to uncertainty and behavior rooted in established hierarchical role understanding. These findings resonate with the studies of Pollard [28] and Thistlethwaite [29], suggesting that the knowledge gap should be addressed by educators and health institutions.

### Adversarial team culture

Related to the role patterns discussed above, our results suggest that factors linked to team culture serve to discourage nurses from assuming responsibility. Vaismoradi *et al*. [30] showed that a perception of insecurity, fear and hierarchy discouraged nurses from taking responsibility. Student statements presented here, mainly categorized under the sub-theme *avoiding responsibility*, suggest that elements of such a work culture still prevail. Nursing students and some medical students had experienced being deterred by conflicts, reproaches, and a sense of being sidelined and alienated. Discussing such behavior, Street [31] introduced the concept of *differential visibility*: “*nurses becoming visible or invisible to others depending on the person, the place, the time* …” (p. 51). Nursing students in the current study expressed reluctance to voice their opinions, and hence became “visible” to the other team members. This pattern of conduct may adversely affect the treatment and care of the patient, especially since nurses observe patients for extended periods of time and may possess information unknown to the rest of the team [25,32,33].

Some of the medical students stated they also had encountered a sense of insecurity in their role performance during the ward rounds.

### Use of interprofessional arenas for learning

Analysis of the students’ statements unveiled a wide variation in the perception of interprofessional arenas, depicting them as venues characterized by collaborative learning, distrust, confrontation, disrespect, and hierarchical structures.

A number of students concluded that the daily rounds – and the corresponding “huddles” – offered preferred arenas for interprofessional teamwork training. The justification for this varied, but rested at least partly on the impression that the daily rounds and “huddles” allowed time for at least a minimum of discussion between team members, although this depended on the chief physician in charge. The purpose of daily rounds was somewhat ambiguous, and some students expressed that a training effort might focus on the clarification and redefinition of its purpose. It was also mentioned that the daily rounds afforded the patient an opportunity to voice concerns. The students’ reasoning on this point is supported by Nikendei *et al.*[34] and Williamson *et al*. [35] who concluded that ward rounds training was urgently required. Nørgaard *et al*. [36] and Weber *et al*. [33] also who suggested that daily rounds should be considered one of the most important arenas for promoting interprofessional training in clinical practice. Caldwell *et al*. [37] and Stew [38] asserted that the best arenas for learning teamwork are characterized by well-established teams that hold regular meetings, and that involve patients in care decisions, criteria that seem to some degree to be consistent with typical daily round procedures.

Several students from both groups advocated psychiatric and rehabilitation wards as arenas conducive to interprofessional teamwork. The underlying psycho-sociological processes are not obvious, but many students associated the psychiatric ward culture with qualities favorable to interprofessional teamwork, and mentioned that they felt “accepted and respected” more than in other wards. This suggests that more research is warranted to untangle what attributes of the psychiatric ward culture that favor teamwork, and to further investigate whether these qualities can be exploited in other arenas.

With few or no student arenas for formal training in teamwork skills, the participants in this study perceived the focus group interviews, themselves, as a valuable arena for knowledge exchange. This suggests that the format of focus group interviews may merit further use in university health care programs and in health institutions.

### Limitations

The present study’s use of a small sample of students prevents these findings from providing an accurate representation of the sentiments of all medical and nursing students at these universities. The study took place at a single clinical training institution in Norway. As a result, the applicability of its findings may be limited.

## Conclusions

Based on focus group interviews with nursing and medical students, the current study demonstrated that interprofessional teamwork is significantly affected by the professional role identities of the participants. Traditional patterns of professional roles is still highly prevalent in health care teams, influencing several aspects of teamwork, including the participants’ predisposition to communicate freely and share responsibility, both of which are considered fundamental pillars of teamwork.

Moreover, our results indicate that medical and nursing students suffer from a lack of mutual knowledge of each other’s competence and capabilities.

The study also found substantial variation in the perception of the various interprofessional teamwork arenas, ranging from arenas favorable to collaborative learning to arenas characterized by distrust, confrontation, disrespect, and hierarchical structures.

When recommending an arena for interprofessional team training, many students advocated for daily rounds and the corresponding “huddles”, or alternatively, a psychiatric ward, options that seem to reflect many of the criteria proposed in previous studies.

## Competing interests

The authors declare that they have no competing interests. The authors alone are responsible for the writing and content of the paper.

## Authors’ contributions

IAA, BSH and KA designed the study. IAA and BSH conducted the data collection and IAA drafted the manuscript. All authors contributed to the data analysis. BSH and KAA provided critically important feedback to improve the manuscript. All authors read and approved the final manuscript.

## Pre-publication history

The pre-publication history for this paper can be accessed here:

http://www.biomedcentral.com/1472-6920/14/170/prepub
